# Mechanisms of resistance to anti-EGFR therapy in colorectal cancer

**DOI:** 10.18632/oncotarget.14012

**Published:** 2016-12-18

**Authors:** Ben Zhao, Lu Wang, Hong Qiu, Mingsheng Zhang, Li Sun, Ping Peng, Qianqian Yu, Xianglin Yuan

**Affiliations:** ^1^ Department of Oncology, Tongji Hospital, Huazhong University of Science and Technology, Wuhan, Hubei Province, China

**Keywords:** colorectal cancer, epidermal growth factor receptor, targeted drug, primary resistance, acquired resistance

## Abstract

Targeting the epidermal growth factor receptor (EGFR) either alone or in combination with chemotherapy is effective for patients with RAS wild type metastatic colorectal cancer (mCRC). However, only a small percentage of mCRC patients are sensitive to anti-EGFR therapy and even the best cases finally become refractory to this therapy. It has become apparent that the RAS mutations correlate with resistance to anti-EGFR therapy. However, these resistance mechanisms only account for nearly 35% to 50% of nonresponsive patients, suggesting that there might be additional mechanisms. In fact, several novel pathways leading to escape from anti-EGFR therapy have been reported in recent years. In this review, we provide an overview of known and novel mechanisms that contribute to both primary and acquired anti-EGFR therapy resistance, and enlist possible treatment strategies to overcome or reverse this resistance.

## INTRODUCTION

Colorectal cancer (CRC) ranks among the third most common human malignant diseases and is one of the leading causes of cancer-related deaths globally [1, 2]. In recent years, new anticancer drugs that target oncogenic signaling pathways have been developed and have demonstrated a prominent efficacy in the treatment of metastatic colorectal cancer (mCRC). Two representative examples of such drugs are cetuximab and panitumumab, two monoclonal antibodies (moAbs) against the epidermal growth factor receptor (EGFR), which have been proven to be effective for patients with *RAS* wild type (RAS-WT) mCRC in randomized clinical trials [3–9]. However, only a small percentage of mCRC patients are sensitive to anti-EGFR therapy [10], and even those who initially respond to the therapy eventually develop resistance to it [11–13]. Numerous studies have been conducted to explore resistance mechanisms to EGFR blockade, and it seems that several biomarkers and pathways are involved in the development of resistance to anti-EGFR therapy. Here, we provide an overview of these potential resistance mechanisms that can facilitate further improvement of anti-EGFR therapies.

**Figure 1:**
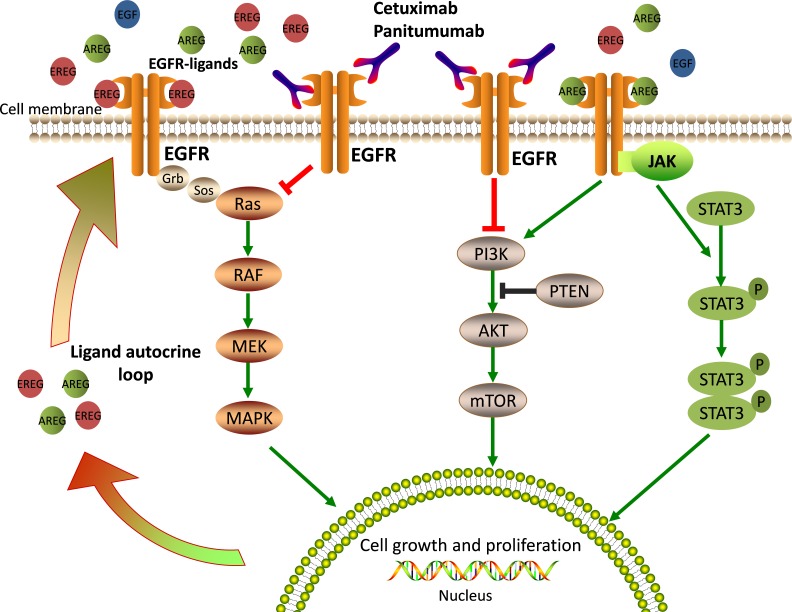
EGFR-mediated signaling pathways and mechanisms of anti-EGFR therapy EGFR ligands bind the extracellular domain of EGFR, lead receptor activation and stimulate downstream signaling pathways that are crucial for cell growth and proliferation. Cetuximab or Panitumumab prevents ligand binding to EGFR, thus blocking EGFR signaling.

EGFR (also called ERBB1/HER1) is a transmembrane receptor tyrosine kinase (RTK) belonging to the ERBB-family. Cetuximab and panitumumab bind to the extracellular domain of EGFR, thereby preventing activation of the receptor tyrosine kinase and of multiple downstream signal transduction cascades that are related to cell survival, proliferation, metastasis, and angiogenesis (Figure 1) [14, 15]. Among the major downstream pathways activated by EGFR, the RAS-RAF-MAPK, PI3K-PTEN-AKT, and JAK/STAT pathways have also been implicated in the resistance mechanisms against antibody-mediated EGFR inhibition [16]. Any alterations in their components, such as KRAS, NRAS, BRAF, and PIK3CA gene mutations, can lead to constitutive activation of EGFR and the ensuing intracellular signaling and ultimately, to drug resistance [17, 18]. In the following sections, we discuss recent research concerning anti-EGFR therapy and present and overview of the possible mechanisms that may contribute to the development of primary and secondary resistance to anti-EGFR therapy in mCRC.

## PRIMARY RESISTANCE TO ANTI-EGFR THERAPY IN CRC

Early studies have demonstrated that about 80% of unselected mCRCs do not benefit from anti-EGFR therapy [2, 19–21], suggesting that primary resistance to anti-EGFR therapy is common in CRC. Consequently, new drugs that target a single EGFR still have great limitations in the treatment of mCRC.

### Alterations in EGFR and EGFR ligands

Alterations of the EGFR, including *EGFR* gene copy number and EGFR-specific ligands, have been considered in recent years, and both have been confirmed to be associated with responses to EGFR inhibitors in retrospective clinical trials [22, 23].

#### Low EGFR gene copy number

It is certain that preclinical and/or clinical studies with an EGFR-targeted agent often demonstrated the complex relationship between EGFR alterations (somatic mutations and gene copy number variations) and the efficacy of the anti-EGFR therapy. In 2004, Thomas et al. found that mutations in EGFR strikingly correlate with the clinical responsiveness to EGFR tyrosine kinase inhibitors (TKI) in patients with non-small-cell lung cancer (NSCLC) [24]. A similar result was observed in the use of the anti-HER2/neu receptor moAb trastuzumab for patients with metastatic breast cancer, in which the degree of HER2 expression correlates with response to trastuzumab [25]. However, mutations in the EGFR kinase domain are an extremely rare event in patients with CRC, and when they do occur, they are not associated with patient response [26]. Therefore, numerous studies were focused on the altered gene copy number of *EGFR* [22, 27, 28].

In a cohort study examining the correlation between *EGFR* gene copy number and clinical response to anti-EGFR therapy [22], about 90% of patients with objective responses after cetuximab or panitumumab treatment showed increased *EGFR* copy number (assessed by fluorescence *in situ* hybridization, FISH). In contrast, only 5% of the non-responders showed an increased *EGFR* copy number. More importantly, these data indicate that almost none of the patients (20 of 21 non-responders) with a low *EGFR* gene copy number could benefit from anti-EGFR therapy. Subsequently, Sartore-Bianchi et al. obtained a similar result in a larger and more homogeneous cohort [27]. Both analyses indicate that *EGFR* gene copy number might contribute to resistance to anti-EGFR therapy. Nevertheless, the degree of EGFR expression does not seem to correlate with effectiveness of EGFR inhibitors, therefore, the mechanism thereby the *EGFR* copy number influences the response to EGFR-targeted drugs remains unknown and requires more exploration. Moreover, due to technical obstacles and considerable discrepancies between scoring systems at present, evaluation of sensitivity to anti-EGFR drugs through estimation of *EGFR* gene copy number is still unpractical in clinical practice [29, 30].

#### Low expression of AREG and EREG

AREG and EREG are EGFR-specific ligands that have a key effect on intracellular signaling and are strongly related with response to anti-EGFR therapy. For example, in a prospective clinical trial of 110 patients with mCRC [23], the *AREG* and *EREG* gene expression levels, which were measured from pre-treatment metastatic biopsies, were found to be associated with cetuximab efficacy. Data from gene expression profiles show that patients with tumors expressing high levels of the EGFR ligands AREG and EREG, are more likely to respond to cetuximab (EREG, *P* = 0.000015; AREG, *P* = 0.000025) when compared with patients showing low expression of these ligands. A later study on larger cohorts described a similar observation in *KRAS* wild type (WT) patients [31]. In this study, the gene expression of both EREG and AREG, as well as the status of KRAS were taken into account. In patients with *KRAS* WT tumors, there was a significant correlation between EREG and AREG expression levels and progression-free survival (PFS) and overall survival (OS). For example, in the high EREG expression group, the median OS was 65 weeks, whereas in the low EREG expression group, it was 31 weeks. Similar data were obtained for AREG. Interestingly, *KRAS* WT patients with low ligand expression essentially respond like those with *KRAS* mutant tumors, and both show the worst response to cetuximab. In summary, low *AREG* and *EREG* gene expression levels correlate with resistance to anti-EGFR therapy. In fact, the expression of AREG and EREG is coordinately regulated, and plays an important role in tumor growth and survival by generating an autocrine loop through EGFR (Figure 1). Low levels of expression of AREG and EREG may characterize a tumor that is less dependent on EGFR and, therefore, particularly prone to develop resistance to EGFR inhibitors.

All the data presented above identify a relationship between *EGFR* copy number and EGFR ligand expression, and outcome. Altogether, these findings clearly demonstrate that anti-EGFR moAbs are likely to work most efficiently against amplified targets.

### RAS mutation

**Figure 2:**
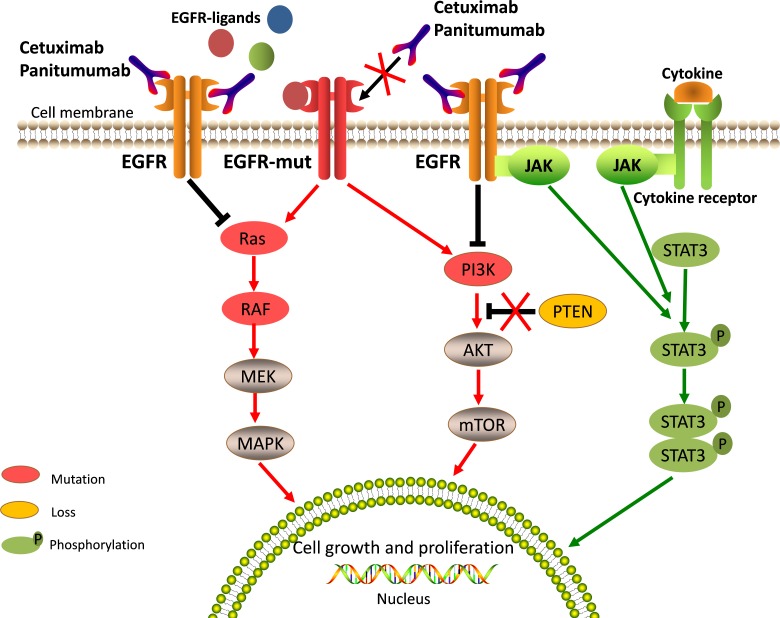
Aberrated genetic alterations in the members of EGFR signaling pathways induce resistance to anti-EGFR therapy Aberrated genetic alterations, including RAS, BRAF, PIK3CA, EGFR S492R mutations, PTEN loss, and STAT3 phosphorylation contribute to the resistance through constitutive activation of EGFR downstream signaling cascades regardless of EGFR blockade. The molecules implicated in EGFR signaling and affected by resistant alterations are highlighted in special colors and described in note.

RAS family genes (including KRAS, NRAS and HRAS) that encode guanosine-5′-triphosphate (GTP)-binding proteins play important roles in EGFR-activated signaling pathways [21, 32]. Activating mutations in RAS are common in CRC. RAS mutations are detected in about 50% of CRC patients; KRAS mutations are found in about 40% of cases, NRAS mutations are found in about 3-5% of cases, and HRAS mutations are negligible events [33, 34]. Mutations in RAS genes often lead to constitutive activation of RAS proteins and RAS downstream effector pathways (Figure 2). Persistent downstream signaling through the RAS axis can activate multiple processes involved in tumor progression and metastasis without the influence of EGFR and other cell surface receptor kinases [32]. Therefore, it can be readily anticipated that mutations in the RAS gene will play a major role in primary resistance to anti-EGFR therapy.

#### KRAS exon 2 mutations

In CRC, about 85-90% of *KRAS* mutations occur in exon 2 (codons 12 and 13) [21, 35]. A large number of retrospective trials over the last decade have strongly suggested that patients with *KRAS* mutations in codon 12 or 13 do not benefit from anti-EGFR therapy [3, 5, 36, 37]. Indeed, *KRAS* exon 2 mutations are by far the most common predictor of resistance to the anti-EGFR drugs cetuximab and panitumumab in patients with mCRC, and have been used to plan appropriately the treatment regimen in clinical practice. All CRC patients are now compulsively tested for seven mutations in the *KRAS* codons 12 and 13 before receiving cetuximab or panitumumab [20].

However, conflicting data exist regarding the codon 13 mutation (G13D) of *KRAS* gene [38, 39]. A large number of retrospective studies indicated that patients carrying the KRAS G13D mutation might derive benefit from treatment with cetuximab [38]. In this analysis, that data of 579 patients with chemotherapy-refractory CRC treated with cetuximab from seven studies were pooled together. Finally, patients with the *KRAS* G13D mutant tumors achieved better OS and PFS in comparison with patients with tumors harboring other *KRAS* mutations (OS: 7.6 VS 5.7mon, HR = 0.50, *P* = 0.004; PFS: 4.0 VS 1.9mon, HR = 0.51, *P* = 0.005). A positive correlation was found between KRAS G13D mutations and OS benefit with cetuximab treatment. Moreover, a further analysis that investigated the updated pooled data sets from the CRYSTAL and OPUS studies designed by Tejpar et al. also shows a similar result [39]. In contrast, a more recent retrospective analysis of 110 patients treated with cetuximab, indicates that patients with KRAS G13D mutations were unlikely to respond to cetuximab [40]. There was no significant difference between patients carrying KRAS G13D mutations and patients with other KRAS mutations in terms of OS and PFS (OS: 8.2 VS 14.6mon, HR = 0.50, *P* = 0.084; PFS: 4.96 VS 3.1mon, HR = 0.88, *P* = 0.72). Consistent with the above analysis, patients with tumors harboring the KRAS G13D mutations were unlikely to benefit from panitumumab therapy in a pooled analysis of three randomized phase III trials [41]. Overall, due to the limitations of the retrospective nature of the present analysis and the low number of patients with such specific mutations, the role of KRAS G13D mutations in the mechanism of primary resistance to anti-EGFR therapies remains controversial.

#### Extended RAS: NRAS and other KRAS mutations

Recently, it has been shown that the mutational status of other RAS family genes outside exon 2 is also associated with response to anti-EGFR therapy [42–45]. In an updated analysis of the randomized phase III CRYSTAL study, the treatment outcomes of patients with *KRAS* exon 2 wild-type tumors were reassessed with respect to additional RAS mutations (*KRAS* exons 3 and 4; *NRAS* exons 2, 3 and 4) [46]. In summary, Van Cutsem et al. found that approximately 15% (63/430 patients) of patients with the *KRAS* exon 2 wild type disease had other RAS mutations. The presence of these extended RAS mutations was associated with poor response to additional use of cetuximab for both PFS (7.2 VS 6.9mon, HR = 0.81, *P* = 0.56) and OS (18.2 VS 20.7mon, HR = 1.22, *P* = 0.50). In contrast, patients with tumors that had no *RAS* mutations showed significantly longer PFS (11.4 VS 8.4mon, HR = 0.56, *P* < 0.001) and OS (28.4 VS 20.2mon, HR = 0.69, *P* = 0.0024) when cetuximab was added to chemotherapy. Thus, similar to findings in patients with *KRAS* exon 2 mutations, additional RAS mutations might also play an important role in anti-EGFR therapy resistance in patients with mCRC.

Furthermore, a recent subset analysis of data from the PRIME trial showed a consistent result that mutations occurring beyond the *KRAS* exon 2 could predict a lack of clinical benefit from Panitumumab administered in combination with first-line chemotherapy [44]. In this study, Doulliard et al. present the negative effects of panitumumab treatment with FOLOFX4 on PFS and OS in patients with tumors bearing mutations in *KRAS* exon 3 (at codon 61) or 4 (at codons 117 and 146), or in *NRAS* exon 2 (at codons 12 and 13), 3 (at codon 61), or 4 (at codons 117 and 146).

Altogether, the current findings clearly show that RAS mutations in exon 2, 3, or 4 (KRAS G13D mutation is still under debate) represent the most important predictive biomarkers of primary resistance to anti-EGFR therapy in mCRC. For this reason, patients with any known RAS mutation should not be treated with either cetuximab or panitumumab.

### BRAF mutations

Although it is clear that the RAS status could help identify a patient population that is unlikely to benefit from anti-EGFR therapy, not all patients with tumors containing wild-type RAS respond to treatment with anti-EGFR therapy. Additional molecular alterations in the downstream components of the EGFR signaling network are also likely to associate with resistance to anti-EGFR moAbs. Among these, BRAF, a downstream effector of RAS in the EGFR pathway, has been a subject of focus. Approximately 5% to 9% of CRC harbor BRAF mutations. More than 95% of these mutations occur in the BRAF V600E allele [47]. The BRAF V600E mutation is able to promote tumor cell proliferation and survival by constitutive activation of the mitogen-activated protein kinase (MAPK) signaling pathway (Figure 2) [48–50]. Moreover, preclinical and clinical studies have suggested that, regardless of EGFR blockade, mutations in *the* BRAF V600E can still lead to persistent activation of downstream signaling resulting in cell proliferation and survival [48–50].

It has become clear that the BRAF V600E mutation is a marker of resistance to anti-EGFR therapy in the chemotherapy-refractory setting. A retrospective trial with clinical data from 11 centers in seven European countries analyzed the effect of BRAF mutations on the efficacy of cetuximab plus chemotherapy in patients with chemotherapy-refractory mCRC [17]. This trial concluded that patients with BRAF V600E mutations showed a significantly lower response rate than those with wild type tumors (8.3% *vs*. 38.0%, OR = 0.15, *P* = 0.0012). A similar result was demonstrated in a more recent multicenter randomized PICCOLO trial [51]. Taken together, these data suggest that the BRAF V600E mutation contributes to resistance to anti-EGFR moAbs in patients with chemotherapy-refractory KRAS wild-type mCRC. However, observations from first-line therapies are not entirely concordant with this conclusion. In a recent retrospective analysis, Van Cutsem et al. showed that carriers of BRAF mutation gained additional benefit from cetuximab in combination with first-line chemotherapy [5].

To further identify the role of BRAF mutations in the response to EGFR inhibition in patients with mCRC, numerous meta-analyses have been carried out. Of these, one recently published study identified 10 randomized controlled trials (including CRYSTAL and OPUS trials) that enrolled 463 patients with BRAF mutations [52]. It shows that the addition of cetuximab or panitumumab treatment did not significantly enhance the benefit of standard therapy or the best supportive care among RAS-wild-type/BRAF-mutated patients in terms of both PFS (HR, 0.88; 95% CI, 0.67-1.14; *P* = 0.33) and OS (HR, 0.91; 95% CI, 0.62-1.34; *P* = 0.63). Overall, it suggests that BRAF mutations are in fact associated with resistance to anti-EGFR therapy. Based on these data, a novel combination therapy of BRAF and EGFR inhibitors was administered in patients with BRAF-mutant CRC, and in some of the cases it resulted in improved response rates [53, 54].

These findings therefore, demonstrate a strong causal relationship between the presence of the BRAF V600E mutation and resistance to anti-EGFR therapy in mCRC. We believe that, similar to KRAS, the BRAF *V600E* mutation could also identify patients that are unlikely to respond to EGFR inhibition. In addition, BRAF mutations are limited to tumors that do not carry the *RAS* mutation [55]. Therefore, consideration of both BRAF and RAS mutations in tumors before administering anti-EGFR therapy can help identify more than half of the non-responders.

### Activation of *PIK3CA/PTEN* signaling pathway

In addition to the RAS/RAF axis, EGFR also triggers the PIK3CA/PTEN signaling pathway. Both these pathways are major downstream pathways of EGFR and can be blocked by EGFR inhibitors, resulting in tumor cell apoptosis [16]. However, molecular alterations of the PIK3CA/PTEN pathway, including active mutations of PIK3CA or the loss of PTEN expression, can lead to activation of downstream signaling pathways through EGFR-independent mechanisms (Figure 2). Therefore, the role of activated PIK3CA/PTEN signaling in the development of EGFR inhibition resistance has been explored.

#### Mutations in PIK3CA exon 20

PIK3CA, a catalytic subunit of class I PI3K, encodes the p110a protein kinase, which is a downstream effector of EGFR. Mutations of PIK3CA are reported in approximately 10-18% of mCRC patients and can coexist with both RAS and BRAF mutations [56, 57]. More than 80% of these mutations occur in exon 9 (E542K, E545K) or exon 20 (H1047R) [17]. PIK3CA mutations lead to constitutive activation of the p110a protein kinase and its downstream signaling pathway, thus resulting in tumor cell proliferation and survival.

In mCRC, several studies have evaluated the potential role of PIK3CA mutations as a predictor of resistance to anti-EGFR therapy [21, 58–60]. However, the results of these studies were highly inconsistent. In order to explain these conflicting results, subtle alterations of PIK3CA were explored. Indeed, biochemical studies show that the PIK3CA mutations in exon 9 and exon 20 have different effects. Exon 9 mutations trigger gain of function through RAS-GTP binding, whereas exon 20 mutations do so independent of interaction with RAS-GTP [61]. Thus, it is reasonable to assume that PIK3CA exon 9 and exon 20 mutations may exert different effects upon treatment with anti-EGFR moAbs. In a large retrospective study that investigated the effect of PIK3CA mutations on the response to cetuximab-based therapy [17], the European consortium found that in the *KRAS* wild-type background, carriers of *PIK3CA* exon 20 mutations showed significantly lower response rates than carriers of wild-type *PIK3CA* (0·0% *vs*. 36·8%; 95% CI 0·00-0·89; *P* = 0·029), whereas exon 9 mutations showed no significant effect (28·6% *vs*. 36·3%; 95% CI 0·25-1·78; *P* = 0·47). Subsequently, the conclusion drawn from a meta-analysis of 13 retrospective cohort studies was that only *PIK3CA* exon 20 mutations were associated with a lack of response to anti-EGFR MoAbs [62]. Overall, it is clear that *PIK3CA* exon 9 mutations and exon 20 mutations differ in their predictive power with respect to anti-EGFR therapy responses.

Based on the review of the current literature, it seems that *PIK3CA* exon *20* mutations are associated with resistance to anti-EGFR moAbs. However, considering the relatively low frequency of occurrence of these mutations, large randomized clinical trials need to be conducted before definitive conclusions can be drawn.

#### PTEN loss

PTEN negatively regulates the PI3K-AKT signaling pathway through its lipid phosphatase activity, and acts as a tumor suppressor gene. PTEN activity can be lost through either *PTEN* gene silencing or mutation [63]. Loss of PTEN expression is estimated to occur in about 20-40% of patients with mCRC [64]. This loss results in constitutive activation of the PI3K-AKT signaling pathway, leading to tumor cell proliferation and survival.

In breast cancer patients, loss of PTEN protein has been identified as a negative predictor of the efficacy of rastuzumab, an anti-HER2 moAb [65]. However, the role of PTEN loss in CRC remains uncertain. Several studies have reported conflicting and inconclusive results on the impact of PTEN loss on anti-EGFR resistance [59, 66, 67]. For example, Sartore-Bianchi et al. showed in 2009, that PTEN loss is associated with decreased response rate (RR), PFS, and OS in a cohort of 110 patients treated with anti-EGFR moAbs [59]. However, in the same year, Laurent-Puig et al. reported that no significant differences were found in terms of RR, PFS, or OS in association with PTEN expression in a larger patient series [66]. Moreover, another study by Loupakis et al. has confirmed that the data on the loss of PTEN expression are not completely concordant between primary tumors and metastases [67]. In this study, one of 22 patients (5%) with PTEN-negative (detected by the IHC method) metastases responded to cetuximab-based treatment, whereas 12 of 33 patients (36%) with PTEN-positive metastases were partial responders (OR, 12.00; 95% CI, 1.43 to 100.75; *P* = 0.007). However, such differences with respect to PTEN expression were not observed in primary tumors.

Since there are numerous differences in the analysis of PTEN expression, including IHC scoring algorithms and inconsistent expression in primary and metastatic tumor samples, loss of PTEN expression cannot be reliably regarded as a negative biomarker of the efficacy of anti-EGFR moAbs. Further investigation and prospective large randomized clinical trials are still required to fully confirm the role of PTEN in anti-EGFR therapy resistance.

### Excess activation of JAK/STAT signaling pathway

The Janus family of tyrosine kinases (JAK) and the signal transducer and activator of transcription (STAT) family are necessary components of cytokine receptor signaling that are actively involved in cellular survival, proliferation, differentiation, and apoptosis. STAT3 is a member of the STAT family of transcription factors that mediate cellular responses to cytokines and growth factors, and is upregulated in many cancers, including CRC [68]. Persistent activation of STAT3, mediated by autocrine and paracrine production of cytokines through the JAK family, as well as activation of tyrosine kinases, such as EGFR and SRC, plays a critical role in oncogenesis, angiogenesis, invasion, metastasis and immune system suppression (Figure 2) [69]. Accumulating evidence supports a role for STAT proteins also in resistance to EGFR inhibitors in several preclinical models, including glioma, head and neck squamous cell carcinoma (HNSCC), and non-small cell lung cancer (NSCLC) [70, 71]. These results provide evidence that STAT3, constitutively activated in CRC, may also play an important role in anti-EGFR treatment resistance.

In a recent study, Qiong Li et al. investigated the mechanism underlying the disappointing effect of the EGFR inhibitor gefitinib in CRC cells, and found that STAT3 phosphorylation (pSTAT3) highly correlated with gefitinib resistance in CRC cells [72]. Their study demonstrates that elevated pSTAT3 levels, mediated by nuclear pyruvate kinase isoform M2 (PKM2), are linked to gefitinib-resistance in CRC cells. Furthermore, inhibition of STAT3 activity by Stattic, a STAT3-specifc inhibitor, or STAT3-specifc siRNA significantly enhanced the efficacy of gefitinib against CRC cells, both *in vitro* and *in vivo*. A similar result was obtained by AS Yar Saglam et al., who demonstrate that combined treatment with cucurbitacin B, a JAK/STAT3 pathway inhibitor, and gefitinib could lead to enhanced antitumor activity in human CRC cells. Therefore, combining EGFR blockade with suppression of JAK/STAT3 signaling is more effective in inhibiting CRC cell growth than inhibition of either pathway alone [73].

These findings suggest that activation of the JAK/STAT3 pathway could contribute to EGFR inhibition resistance in CRC, and targeting the STAT3 pathway may enhance the antitumor effects of EGFR inhibitors and therefore abrogate anti-EGFR therapy resistance.

### Epithelial-to-mesenchymal transition (EMT)

EMT is a complex biological process wherein epithelial cells procedurally lose their original morphology and simultaneously acquire mesenchymal characteristics [74]. EMT enhances the motility and invasion potential of cells and contributes to a number of cancer-related events, including cancer invasion, metastasis, and treatment resistance [75]. Previous research suggests that the EMT-like transitions that occur in carcinoma cells attenuate the role of EGFR signaling in regulating cell proliferation and survival [76]. These studies consider EMT as a kinase switching mechanism, which, in case of EGFR kinase blockade, leads to signaling activation through alternative tyrosine kinases. Therefore, an EMT-like transition has been implicated as a potential mechanism of anti-EGFR therapy resistance.

In a preclinical analysis, Buck et al. observed a strong correlation between E-cadherin (epithelial marker) expression and growth inhibition by EGFR inhibitors in CRC cells [77]. Accordingly, epithelial cell lines showed 7-fold more sensitivity to an EGFR inhibitor compared to mesenchymal-like CRC cells. Moreover, the resistance of mesenchymal-like CRC cells to anti-EGFR drugs could be overcome upon combined inhibition of EGFR and CRIPTO, an important signaling node that induces EMT [78]. Overall, the data support a possible role of EMT as a mediator of resistance to anti-EGFR drugs in CRC cells.

However, are cellular mesenchymal-like alterations generated during the course of anti-EGFR treatment, and do they contribute to acquired resistance as well? To answer this question, Sandra Schmitz et al. extended their study to compare pre- and post-cetuximab tumor biopsies for gene and protein expression [79]. As a result, both gene expression profile analysis and quantitative real-time PCR showed significantly increased expression of the known EMT markers *LEF1*, *TWIST1*, and *ZEB1* in post-treatment biopsies compared with pre-treatment biopsies. This study demonstrates that anti-EGFR treatment could promote EMT, and consequently, contribute to the development of resistance to the treatment itself.

In the phase III randomized TRIBUTE trial, EMT was proven to be related with insensitivity to erlotinib in patients with NSCLC [80]. However, there is no clinical evidence for EMT-induced resistance to EGFR inhibitors in mCRC. Indeed, compared with lung cancer, the current research on EMT-induced resistance in mCRC is very limited. There are still many questions related to EMT-induced resistance in mCRC that need to be answered, such as: 1) What are the mechanisms of EMT-induced resistance? and 2) Can the preclinical studies translate into clinical practice? Although limited data are available for mCRC, we believe that these lacunae will be filled by further evaluation in future studies. Furthermore, an understanding of EMT related resistance to EGFR targeting could provide novel therapeutic opportunities for CRC treatment.

## ACQUIRED RESISTANCE TO ANTI-EGFR THERAPY IN CRC

Nearly all patients with mCRC that initially respond to EGFR monoclonal antibodies eventually show disease progression. This progression upon anti-EGFR therapy is known as acquired resistance. Clinical data indicate that response to anti-EGFR therapies is relatively short-lived and most tumors become refractory within 3-12 months [81]. It is therefore conceivable that numerous mechanisms might contribute to this acquired resistance to anti-EGFR antibodies.

### Secondary alterations in the RAS/RAF signaling pathway

The RAS/RAF signaling axis is one of the most important downstream signaling pathways of EGFR and has been highlighted by its role in primary resistance to anti-EGFR therapy in mCRC. Indeed, genetic alterations in RAS/RAF signaling are also the most common molecular mechanism that drives secondary resistance.

KRAS mutations, in addition to being a key driver of primary resistance to anti-EGFR antibodies in CRC, play a vital role in acquired resistance as well. Approximately 50% of acquired resistance cases occur due to secondary KRAS mutations [82, 83]. Both pre-clinical models and clinical samples have proven that the emergence of KRAS mutations is a mediator of acquired resistance to EGFR inhibitors. In 2010, Bouchahda et al. reported the first case of CRC liver metastasis, wherein tumor KRAS mutations were detected after the development of resistance to cetuximab [84]. In this case, no KRAS mutation was detected in the primary or metastatic tumor samples before the beginning of the cetuximab treatment. However, upon cetuximab treatment, further liver relapse occurred, and two KRAS mutations at codon 13 and 12 were detected in the metachronous liver metastatic tissues. A similar study was subsequently performed by Misale et al., who analyzed the molecular profiles of relapsed tumors from CRC patients [83]. Six of the 10 patients that were KRAS wild type prior to treatment showed KRAS mutations in their plasma samples while receiving cetuximab. In contrast, KRAS mutations were not detected in patients who underwent chemotherapy alone. Interestingly, in the same study, Misale et al. also found one relapsed case, where the patient receiving anti-EGFR moAbs exhibited *KRAS* amplification, which is an otherwise infrequent event in CRC. It is therefore clear that the emergence of KRAS mutations and *KRAS* amplification is associated with acquired resistance to EGFR inhibitors. Likewise, secondary mutations in NRAS and BRAF are also associated with secondary resistance. For instance, in a preclinical model, cetuximab- and/or panitumumab-resistant CRC cell lines, initially sensitive to anti-EGFR moAbs, developed resistance after continuous anti-EGFR treatment. Strikingly, in addition to the alterations at the known hotspots of the *KRAS* gene, NRAS and BRAF mutations were also found in the resistant populations and also in some cell lines harboring multiple mutations [85].

However, this emergence of “acquired genetic alterations” in RAS/RAF signaling raises the question of whether these alterations are novel spontaneous mutations or whether they are selected from pre-existing resistant subclones by the anti-EGFR therapy. Misale et al. and Diaz et al. addressed this question following different approaches, either *in vitro* or *in vivo* [82, 83]. Strikingly, both groups arrived at a similar explanation namely, that “acquired” alterations in KRAS could be an expansion of pre-existing resistant clones under the pressure of anti-EGFR moAbs. Furthermore, given the existence of inter- and intra-tumor heterogeneity, the explanation of latent resistant clones seems more convincing. In conclusion, acquired RAS and BRAF genetic alterations have been identified as a mechanism of acquired resistance to anti-EGFR therapy in CRC and these genetic alterations most likely arise because of clonal selection of pre-existing resistant cells.

### Activation of alternative growth factor receptor pathways

**Figure 3:**
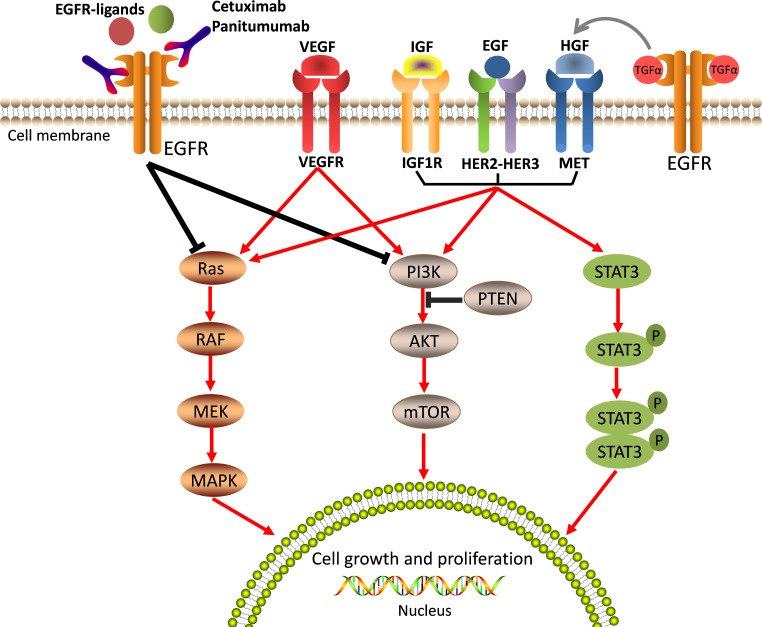
Aberrated activations of the bypass pathways induce resistance to anti-EGFR therapy EGFR downstream effectors can be activated by alternative and/or compensatory membrane growth factors, includingIGF-1R, MET, HER2, and VEGFR. These growth factors then trigger intracellular signaling pathways bypassing EGFR and induce tumour cell growth and proliferation, and lead resistance to anti-EGFR therapy.

The other major mechanism of acquired resistance to anti-EGFR moAbs is the activation of growth-factor signaling pathways by upregulation of alternative and compensatory signaling cascades through receptors other than EGFR. For example, numerous growth factor receptors, such as type 1 insulin-like growth factor receptor (IGF-1R), mesenchymal-epithelial transition factor receptor (MET receptor), and the human epidermal growth factor receptor-2 (HER2) [86–88], can activate EGFR downstream effectors and trigger the ensuing intracellular signaling pathways by bypassing EGFR, thus inducing tumor cell proliferation and resistance to apoptosis (Figure 3).

#### Activation of the IGF-1R pathway

IGF-1R belongs to a family of transmembrane tyrosine kinases. IGF-1R is activated upon insulin-like growth factor (IGF) 1 or IGF-2 binding, and leads to downstream activation of the RAS/RAF/MAPK and PI3K/AKT pathways [86, 89]. In addition, strong molecular cross talk between the IGF-1R and EGFR networks has been demonstrated during recent years. Preclinical studies have shown that activated signaling through IGF-1R leads to increased activation of EGFR [90–92]. Therefore, the effect of anti-EGFR therapy may be bypassed through the activation of alternative, IGF-1R-induced pathways. For instance, in breast cancer, increased activation of the IGF-1R/PI3K/AKT pathway has been found in an anti-EGFR agents and linked with acquired resistance to anti-EGFR moAbs [93]. Similarly, recent studies have implicated IGF-1R and its ligands (IGF-1/IGF-2) in acquired resistance to anti-EGFR therapy in CRC [92, 94]. In a retrospective study of 168 patients with KRAS wild-type mCRC, the expression of IGF1 was evaluated in terms of clinical outcome in patients treated with irinotecan and cetuximab [94]. The result showed that a numerically lower response rate (22% *vs*. 65%; HR, 4.2; 95% CI, 2.0-10.2; *P* = 0.003) was seen in IGF-1-positive groups compared to IGF-1-negative groups. This significant difference demonstrates that increased activation of the IGF-1R pathway is correlated with resistance to anti-EGFR therapy.

As a potential biomarker of resistance to anti-EGFR agents, IGF-1R has recently emerged as an attractive therapeutic target. Preclinical data have demonstrated that combined inhibition of IGF-1R and EGFR resulted in an enhanced anti-tumor effect in xenograft models [95]. Although in numerous early clinical analyses neither IGFR inhibitors alone nor the combination with anti-EGFR moAbs showed any promising anti-tumor activity in patients with anti-EGFR moAb-refractory mCRC [96], in a more recent randomized phase II/III study, a response to the IGF-1R inhibitor was identified by further exploratory biomarker analyses [97]. In this study, 344 eligible patients with *KRAS* wild-type tumors were randomly assigned to dalotuzumab (IGF1R inhibitor) or placebo in combination with cetuximab and irinotecan. The results showed that the addition of dalotuzumab did not improve PFS or OS compared to the placebo group. However, when the effect of the treatment was further evaluated with respect to IGF-1 expression, improvements in PFS (5.6 *vs*. 3.6 months; HR = 0.59; 95% CI = 0.28 to 1.23; *P* = 0.16) and OS (17.9 *vs*. 9.4 months; HR = 0.67; 95% CI = 0.31 to 1.45; *P* = 0.31) were observed in IGF-1 positive tumors compared to IGF-1 negative tumors when dalotuzumab was added. In contrast, the placebo group showed shorter PFS (3.6 *vs*. 6.6 months; HR = 2.15; 95% CI = 1.15 to 4.02; *P* = 0.02), and OS (9.4 *vs*. 15.5 months; HR = 2.42; 95% CI = 1.21 to 4.82; *P* = 0.01) in patients with IGF-1 positive tumors. These synergistic anti-tumor effects further support a role for IGF-1R as a mediator of resistance to anti-EGFR agents.

In conclusion, the IGF system has been attributed an important role in the mechanisms of anti-EGFR therapy resistance in CRC. However, in order to establish the reliable use of IGFR inhibitors in specific anti-EGFR resistant patients a more comprehensive analysis of the existing data is required.

#### MET overexpression and amplification

The *MET* oncogene encodes the tyrosine kinase receptor for Hepatocyte Growth Factor (HGF) and leads to cell proliferation and survival through the activation of intracellular signaling cascades including the PI3K/AKT, RAC1/cell division control protein 42 (CDC42), RAP1 and RAS/MAPK pathways [98]. In NSCLC, activation of MET/PI3K/AKT signaling has been identified as one of the key compensatory pathways to escape the inhibitory effects of the EGFR TKI gefitinib [99, 100]. Moreover, a complex cross-signaling network between EGFR and MET has also emerged in the past few years [98, 101]. EGFR-MET interactions with subsequent activation of the MET pathway induced by the overexpression of TGF-α have been proposed to be a probable mechanism for acquired resistance to cetuximab in CRC cells [101]. These results highlight a possible role for MET in mediating resistance to anti-EGFR therapies in CRC. Indeed, in 2011, Liska et al. demonstrated that HGF-induced MET activation could confer cetuximab resistance to CRC cells [102]. In particular, they showed that HGF-mediated MET activation could rescue CRC cells from cetuximab-induced apoptosis or cell cycle arrest by restoring signaling through the AKT and MAPK pathways. Interestingly, further analysis showed that the effects of cetuximab could again be restored by both pharmacological inhibition and silencing of MET [102].

Furthermore, in an *in vivo* study, analysis of tumor tissues from patients who developed resistance to cetuximab or panitumumab showed the emergence of *MET* amplification in more than 40% (3 out of 7) of the cases [87]. Additionally, *MET* amplification was rarely found in pre-treatment tumor tissues. Only one of the three cases with post-treatment *MET* amplification revealed a rare *MET* amplification in the pre-treatment samples as well. In conclusion, the emergence of MET amplification correlates with acquired resistance to anti-EGFR therapies in CRC, and possibly arises from an expansion of pre-existing *MET* amplified clones under the pressure of anti-EGFR therapy.

Notably, *MET* amplification could also be responsible for primary resistance to anti-EGFR moAbs in CRC. Data obtained from both patient samples and xenografts have identified that amplification of *MET* correlates with a negative response to cetuximab [87, 103]. However, *MET* amplification was found only in about 1% of untreated mCRC cases [87]. For this reason, *MET* amplification cannot be reliably considered as a biomarker of primary resistance to anti-EGFR therapy in mCRC.

#### HER2 amplification and overexpression of the HER3/4 ligand heregulin

HER2 is a member of the HER family of receptor tyrosine kinases that has the ability to activate the MAPK and PI3K/AKT pathways through heterodimerisation with EGFR or HER3 [88, 104]. HER2 leads to the activation of a signaling pathway shared with EGFR and is therefore, a potential biomarker of resistance to anti-EGFR therapy.

In an early analysis, Bertotti et al. took advantage of a large collection of patient-derived mCRC xenografts (‘xenopatients’) to assess the role of the *HER2* gene in cetuximab resistance [105]. Analysis of genotype-response correlations in *HER2*-amplified xenopatients demonstrated that *HER2* gene amplification was specifically related to cetuximab resistance. In addition, this resistance could be overcome through the administration of a HER2 inhibitor. However, a later study by Yonesaka et al. did not completely agree with these findings [104]. Yonesaka et al. found that the effect of HER2 in the resistance to anti-EGFR moAbs was not only limited to the amplification of the receptor, but also to the secretion of the ligand heregulin. In their study with clinical samples, patients with *HER2* gene amplification or overexpression of the HER3/4 ligand heregulin were associated with a significantly poorer PFS and OS. Furthermore, both of these mechanisms could lead to persistent activation of ERK signaling, thus circumventing the anti-tumor effects of anti-EGFR therapy. Both Bertotti et al. and Yonesaka et al. suggested a synergistic anti-tumor effect of the combined inhibition of HER2 and EGFR. Their studies demonstrated that combinations of selective inhibitors targeting HER2 and EGFR were able to significantly inhibit the growth of cetuximab-resistant CRC cells, and induce long-lasting tumor regression in experimental models [104, 105]. These results not only emphasized *HER2* gene amplification and heregulin overexpression as important mechanisms of resistance to anti-EGFR therapy, but also highlighted a possible new therapeutic target for clinical use.

Indeed, *HER2* amplification has been suggested as both an intrinsic, as well as an acquired mechanism of resistance. However, it should be stressed that the prevalence of *HER2* amplification is infrequent in CRC, and occurs only in about 2% of unselected mCRC [104, 106]. Therefore, considering its low frequency, *HER2* amplification is not likely to be a key player in primary resistance to anti-EGFR therapy. On the other hand, pre-existing infrequent *HER2*-amplified clones might be expanded under the selective pressure of anti-EGFR therapy, leading to disease progression. In this regard, *HER2* amplification is more likely to confer acquired anti-EGFR therapy resistance.

### EGFR S492R mutation

It is well known that the T790M mutation of EGFR plays a critical role in acquired resistance to EGFR TKIs in NSCLC. Statistically, more than half of NSCLC patients with acquired resistance to TKIs were found to carry the EGFR T790M mutation [107, 108]. It is thus anticipated that secondary mutations in EGFR might also lead to resistance against anti-EGFR moAbs in CRC. In fact, in 2011, Montagut and colleagues discovered an acquired mutation in the extracellular domain of EGFR (S492R) and proved its association with acquired resistance to cetuximab in mCRC [109]. In an analysis of ten patients that showed disease progression after cetuximab treatment, two patients were detected with the S492R mutation. Moreover, mutations at this site were not detected in their pre-treatment biopsies. *In vitro* findings in a cetuximab-resistant CRC cell model showed a similar result.

Further analysis indicated that the substitution of serine to arginine at amino acid 492 (S492R) is caused either by the 1476C > A or the 1474A > C mutation in the gene region encoding for the extracellular domain of EGFR [109]. This mutation reduces the affinity of the receptor to the ligand and interferes with binding to cetuximab. Notably, the S492R mutation does not inhibit binding of panitumumab to EGFR. Indeed, in the study by Montagut et al., one of the two cetuximab-resistant patients with tumors harboring the S492R mutation responded to subsequent treatment with panitumumab. Therefore, following disease progression upon cetuximab treatment, treatment with panitumumab appears to be a rational strategy for patients harboring the S492R mutation.

Strikingly, in the same study, the S492R EGFR mutation was not detected in any of the tumor samples collected from 156 mCRC patients that did not undergo any therapy. Esposito et al. obtained the same result in a larger cohort of patients [110]. Overall, these findings strongly identified the S492R mutation as a mechanism of acquired but not primary resistance to cetuximab.

### Alteration of VEGF signaling

Vascular endothelial growth factor (VEGF) is a potent signaling molecule that plays a central role in angiogenesis. VEGF binds to and activates three structurally similar receptor tyrosine kinases: VEGFR1 (also known as FLT1), VEGFR2 (also known as KDR), and VEGFR3 (also known as FLT4) [111]. These receptors primarily mediate changes within vasculature, including endothelial cell proliferation and permeability. In addition, VEGF signaling has several important functions that are independent of neovascularization, such as effects on tumor cell survival, migration, and invasion (Figure 3) [112].

Furthermore, aberrant VEGF signaling has been shown to be associated with acquired EGFR inhibitor resistance. Ciardiello et al. demonstrated ten years ago, that elevated expression of VEGF in colon cancer cells was correlated with the resistance to the EGFR inhibitor [113]. In addition, a more recent study by Bianco et al. showed that VEGF, as well as VEGFR1 were secreted at higher levels in cetuximab-resistant cells than in the parental cetuximab-sensitive cells [114]. Moreover, the growth and migration of EGFR inhibitor-resistant cells could be inhibited by VEGFR1 silencing or by vandetanib, an orally available TKI that inhibits EGFR, VEGFR1, and VEGFR-2 tyrosine kinases. These findings indicate that the combined inhibition of VEGFR and EGFR results in restoration of sensitivity to anti-EGFR drugs, and further supports an association between increased expression of VEGF/VEGFR1 and anti-EGFR treatment resistance.

Overall, preclinical experiments have demonstrated that VEGF signaling plays an important role in anti-EGFR therapy resistance and the combination of VEGFR and EGFR inhibitors has been associated with improved anti-tumor activity in xenografts [113]. For these reasons, it was assumed that blockade of VEGF signaling pathway could be a way to overcome anti-EGFR therapy resistance. However, this putative preclinical strategy was not successful in the clinic [115–117]. In the CAIRO2 and PACCE clinical trials, the combination of the anti-VEGF moAb bevacizumab, and the anti-EGFR moAb cetuximab or panitumumab did not result in improved PFS or OS [115, 116]. These results raise the possibility of a negative interaction between anti-EGFR moAbs and anti-VEGF moAbs when combined with chemotherapy in clinical practice. An increase in drug related toxicity is a likely cause of the reduction in survival, since it contributed to increases in dose delays, decreases in dose intensity, and increases in mortality in the dual EGFR/VEGF inhibition arm. In addition, several studies suggest that there potentially exists a negative pharmacodynamic interaction between anti-EGFR moAbs and anti-VEGF moAbs [116]. Therefore, further research is still required in order to develop a more comprehensive understanding of the role of VEGF signaling in the resistance of anti-EGFR therapy.

## OVERCOMING RESISTANCE TO ANTI-EGFR THERAPY

It is clear that aberrant biomarkers, including RAS mutations, BRAF mutations, PIK3CA mutations, PTEN loss, STAT3 phosphorylation, IGF1R activation, *MET* amplification, *HER-2* amplification, and altered VEGF and VEGFR signaling (Table 1) result in resistance to anti-EGFR therapy mainly through constitutive activation of EGFR downstream signaling pathways regardless of EGFR blockade. Consequently, it is reasonable to anticipate that knockdown or inhibition of the resistance pathways will be an effective way to restore sensitivity to EGFR inhibition.

**Table 1:** Genetic and histologic evidence for resistance to anti-EGFR drugs in CRC

Reference/study	Patients included in analysis, n	Study type	Genetic and histologic evidence
Low EGFR gene copy number			
Moroni et al. [22] Sartore-Bianchi et al. [27]	31 92	Clinical study Clinical study	Low EGFR gene copy number was significantly associated with non-response after treatment with cetuximab or panitumumab (with or without chemotherapy). Low EGFR gene copy number was significantly associated with non-response and shorter PFS and OS after treatment with panitumumab.
Low expression of AREG and EREG			
Khambata-Ford et al. [23] Jacobs et al. [31]	110 220	Clinical study Clinical study	Low expression of AREG and EREG was significantly associated with non-response and shorter PFS and OS after treatment with cetuximab. Low expression of AREG and EREG was significantly associated with non-response and shorter PFS and OS after treatment with cetuximab plus irinotecan.
EGFR S492R mutation			
Montagut et al. [109]	10	Preclinical and clinical study	Acquired EGFR ectodomain mutation (S492R) prevents cetuximab binding and confers resistance to cetuximab in human mCRC cell line DiFi. Two of ten individuals with mCRC with disease progression after cetuximab treatment acquired S492R mutation .
RAS mutation			
Allegra et al. [37]a Allegra et al. [35]b	- -	Clinical study Clinical study	KRAS exon 2 (codon 12 and 13) mutations were significantly associated with non-response and shorter PFS and OS in mCRC patients treated with cetuximab or panitumumab (with or without chemotherapy). RAS mutations in exons 2 (codons 12 and 13), 3 (codons 59 and 61), and 4 (codons 117 and 146) of both KRAS and NRAS were associated with non-response and shorter PFS and OS in mCRC patients treated with cetuximab or panitumumab (with or without chemotherapy).
BRAF V600E mutation			
De Roock et al. [17] Rowland et al. [52]	1022 463	Clinical study Meta-analysis	BRAF V600E mutation was significantly associated with a low RR in mCRC patients treated with cetuximab plus chemotherapy. BRAF mutation was significantly associated with shorter PFS and OS after treatment with cetuximab or panitumumab (with or without chemotherapy).
PIK3CA exon 20 mutation			
De Roock et al. [17]	1022	Clinical study	PIK3CA exon 20 mutations were significantly associated with nonresponse and shorter PFS and OS after treatment with cetuximab plus chemotherapy.
PTEN loss			
Sartore-Bianchi et al. [59] Laurent-Puig et al. [66]	110 102	Clinical study Clinical study	PTEN loss was significantly associated with decreased RR, PFS, and OS in mCRC patients treated with panitumumab or cetuximab (with or without chemotherapy). PTEN expression was not significantly associated with RR, PFS, or OS in mCRC patients treated with cetuximab plus chemotherapy.
STAT3 phosphorylation			
Li et al. [72]	-	Preclinical study	Elevated phospho-STAT3 levels correlate with geftinibc resistance in CRC cells and are regulated by nuclear PKM2.
Activated IGF1R			
Scartozzi et al. [94]	168	Clinical study	Elevated expression of IGF1 was significantly associated with lower RR and shorter PFS and OS after treatment with cetuximab plus irinotecan.
MET amplification			
Liska et al. [102] Bardelli et al. [87]	- 7	Preclinical study Preclinical and clinical study	HGF-induced MET activation could confer resistance to cetuximab in CRC cells. MET amplification is associated with primary resistance to cetuximab in CRC patient-derived tumor xenografts. MET amplification is associated to acquired resistance to cetuximab or panitumumab in mCRC patients.
HER2 amplification			
Yonesaka et al. [104] Bertotti et al. [105]	303 -	Preclinical and clinical study Preclinical study	HER2 gene amplification or overexpression of the HER3/4 ligand, heregulin, was significantly associated with lower RR and shorter PFS and OS after treatment with cetuximab (with or without chemotherapy). HER2 gene amplification was specifically related with non-response to cetuximab in CRC patient-derived tumor xenografts.
Altered VEGF/VEGFR			
Ciardiello et al. [113] Bianco et al. [114]	- -	Preclinical study Preclinical study	VEGF was found increased secretion in EGFR inhibitor–resistant CRC cells. VEGF as well as VEGFR1 was secreted at higher levels in cetuximab-resistant CRC cells compared with the parental cetuximab-sensitive CRC cells.
EMT			
Buck et al. [77]	-	Preclinical study	The occurrence of EMT was associated with erlotinibc resistance in CRC cells.

Abbreviation: PFS: progression-free survival, OS: overall survival, RR: response rate, PKM2: pyruvate kinase isoform M2, HGF: hepatocyte growth factor, EMT: epithelial-mesenchymal transition.a: analysis from five single-group studies and five randomised clinical trials. b: analysis from 11 systematic reviews with meta analyses, two retrospective analyses, and two health technology assessments based on a systematic review. c: EGFR kinase inhibitor.

Therefore, a rational approach to block the resistance pathways is simultaneous or sequential targeting of the aberrant biomarkers. Indeed, for almost all the biomarkers that are correlated with resistance, there is already a promising targeted strategy proposed either in preclinical studies or in clinical trials (Table 2). Sorafenib, a potent inhibitor of the V600E B-RAF protein, which is also a well-known multi-targeted kinase inhibitor, has shown pronounced activity in combination with cetuximab in V600E BRAF-mutant CRC cells [118]. In this study, Di Nicolantonio et al. showed that treatment with cetuximab alone was less effective in V600E BRAF-mutant cells than in BRAF-wild-type cells. However, sensitivity to cetuximab in V600E BRAF-mutant cells could be restored by a combination of cetuximab and sorafenib. Furthermore, Al-Marrawi et al. reported a successful clinical outcome with this combination treatment [54]. In their report, the combination of sorafenib and cetuximab resulted in disease stabilization for a period longer than 7 months in a patient with BRAF-mutant mCRC, whose disease had earlier shown resistance to cetuximab. Similar results were found with combination treatments based on inhibitors of other biomarkers related to anti-EGFR drug resistance [119–121].

**Table 2:** Overview of molecular mechanism of resistance and putative strategy to overcome resistance

Genetic alterations	Primary resistance	Acquired resistance	Possible strategy to overcome resistance	Reference
Altered EGFR	Yes	Yes	MEK inhibitors with PI3K inhibitors or mTOR inhibitors; Panitumumaba (EGFR S492R mutation)	[109, 119]
RAS mutation	Yes	Yes	Anti-EGFR with MEK inhibitors	[85, 119, 123]
BRAF V600E mutation	Yes	Yes	Anti-EGFR with BRAF inhibitors or MEK inhibitors	[54, 118, 122]
PIK3CA exon 20 mutation	Yes	Not Sure	Anti-EGFR with PI3K inhibitors or mTOR inhibitors	[119, 120]
PTEN loss	Not Sure	Not Sure	Anti-EGFR with PI3K inhibitors or mTOR inhibitors	[119, 120]
STAT3 phosphorylation	Yes	Yes	Anti-EGFR with STAT3 inhibitors	[72, 73]
Activated IGF1R	Minor Effect	Yes	Anti-EGFR with IGF1R inhibitors	[97]
MET amplification	Minor Effect	Yes	Anti-EGFR with MET inhibitors	[98]
HER2 amplification	Minor Effect	Yes	Anti-EGFR with HER2 inhibitors	[105, 121]
Altered VEGF/VEGFR	No	Yes	Anti-EGFR with anti-VEGF or anti-VEGFR	[113]

**Abbreviation** MEK: (also called MAP2K) mitogen-activated protein kinase kinase, mTOR: mammalian target of rapamycin.

**Superscript** a: treatment with panitumumab is a rational strategy to overcome cetuximab resistance caused by the EGFR S492R mutation.

Another potential way to counteract the resistance pathways is to target essential effectors of EGFR that are downstream of the resistance-related biomarkers, such as mitogen-activated protein kinase kinase (MEK or MAP2K) and mammalian target of rapamycin (mTOR). MEK and mTOR are downstream effectors of BRAF and PI3K, respectively, and potent inhibitors of both have been used in clinical trials. Dual EGFR and MEK and/or mTOR inhibitors showed improved response in tumor models harboring aberrant biomarkers, such as RAS, BRAF and PIK3CA mutations [119, 120, 122, 123], suggesting that they might prevent the activation of resistance pathways. Therefore, rational combinations of targeted treatments that aim at blocking every possible signaling pathway are optimum approaches to reverse anti-EGFR therapy resistance (Table 2).

In fact, there are significant differences in the performance of drugs with respect to resistance development in patients due to tumor heterogeneity, each requiring an individual, and often an uncrossed therapeutic strategy. For example, when resistance to cetuximab is caused by the *EGFR* S492R mutation, subsequent treatment with the alternative EGFR inhibitor, panitumumab, can lead to transient tumor regression [109]. Therefore, to overcome resistance and to prolong the efficacy of EGFR-targeted therapies, it is important to prepare comprehensive strategies based on the mechanisms of resistance in each individual CRC patient. As further steps toward personalized treatment of CRC patients have been taken in the past few years, we predict that additional therapeutic schemes might arise.

## CONCLUSIONS

It is apparent that multiple mechanisms of anti-EGFR therapy resistance exist in CRC, which range from molecular alterations to histological transformations (Table 1). Most of them act individually or in concert to counteract the activity of anti-EGFR drugs. However, resistance to anti-EGFR moAbs is mainly mediated through the constitutive activation of EGFR downstream signaling cascades that can result from either genetic alterations in the members of RAS/RAF, PIK3CA/PTEN, and JAK/STAT pathways (Figure 2) or from the activation of alternative growth factor receptors, such as IGF1R, HER2, and MET (Figure 3). Altogether, these primary mechanisms of resistance account for over 70% of the cases that are unresponsive to anti-EGFR therapies [87].

Although these data are exciting and open new approaches for selecting patients likely to develop insensitivity to anti-EGFR drugs, only RAS mutations are currently approved for clinical consideration. Therefore, numerous retrospective and prospective clinical trials are required to assess whether research on other biomarkers can be translated into effective clinical practice. At the same time, a comprehensive understanding of resistance mechanisms through studies in both preclinical models and CRC patients, will ultimately lead to the development of more effective targeted strategies. Our current understanding of the mechanisms of resistance to anti-EGFR therapies is not yet complete, as additional resistance mechanisms may be undiscovered. As the field of molecular targeting treatment continues to evolve, a more comprehensive picture of resistance mechanisms will form, which will help the development of novel strategies to overcome both primary and acquired resistance.
